# Editorial: Biomechanical and biochemical regulation of the musculoskeletal system

**DOI:** 10.3389/fbioe.2023.1192638

**Published:** 2023-05-05

**Authors:** Jun Pan, Damien Lacroix, Yilu Zhou, Bin Wang

**Affiliations:** ^1^ Bioengineering College, Chongqing University, Chongqing, China; ^2^ Department of Mechanical Engineering, The University of Sheffield, Sheffield, United Kingdom; ^3^ Department of Orthopaedic Surgery, University of Pennsylvania, Philadelphia, PA, United States; ^4^ Institute of Life Sciences, Chongqing Medical University, Chongqing, China

**Keywords:** musculoskeletal system, biomechanical regulation, musculoskeletal disorders, computational model, biochemical regulation

The musculoskeletal system is an essential part of the human body that is responsible for providing support, movement, and protection. The system is composed of bones, muscles, and connective tissues that work together in a complex manner to facilitate the functioning of the body. Over the years, extensive research has been conducted to understand the biomechanical and biochemical regulation of this system, and how these processes are interrelated. The 2017 Global Burden of Disease Study reports that the burden of musculoskeletal disorders accounts for 5.6% of lost years of “healthy” life worldwide (GBD, 2017 DALYs and HALE Collaborators, 2018). This Research Topic in Frontiers in Bioengineering and Biotechnology includes 20 peer-reviewed publications that delve into various aspects of this area of study.

Several factors influence the biomechanical regulation of the musculoskeletal system, including muscle strength, muscle flexibility, joint stability, and joint range of motion (ROM). By understanding these factors, researchers can develop interventions to improve the overall function of the musculoskeletal system. Ching et al. focused on simulating the mechanical and recurrent sprain injuries in chronic ankle instability (CAI) patients, and established a new ankle instability model with multiple ankle injuries using a self-designed machine to sprain the ankle with a controlled inversion angle and speed. This model provided a better understanding of the mechanical factors that contribute to ankle instability, and highlighted that multiple mechanical sprains are a good model for investigating the mechanisms of CAI induced by recurrent sprain injuries. Li et al. explored the biomechanical analysis of oblique-pulling manipulation in treating sacroiliac joint (SIJ) dysfunction. This study showed that pubic symphysis is essential to restrict SIJ motion, and the oblique-pulling manipulation could cause a weak nutation and separation of SIJ. The study highlighted the effects of stretching and loosening of surrounding ligaments in treating SIJ dysfunction. Han et al. performed the biomechanical and clinical study of rod curvature in single-segment posterior lumbar interbody fusion. This study evaluated the effect of rod contouring on single-segment posterior lumbar interbody fusion using the finite element method and retrospective study. The findings of the study have significant implications for the design of spinal implants and the treatment of spinal disorders. Zhang et al. evaluated the biomechanical effects of a novel anatomic titanium mesh cage for single-level anterior cervical corpectomy and fusion using finite element analysis. This study demonstrated the potential benefits of this implant in decreasing the risks of titanium mesh cage subsidence, instrument-related complications, and adjacent segment degeneration after surgery. The study highlighted the importance of biomechanical regulation in the design of cervical spinal implants. Zou et al. focused on the distal humeral trochlear geometry associated with the spatial variation of the dynamic elbow flexion axis. They investigated the relationship between distal humeral trochlear geometry and *in vivo* spatial variation of the dynamic flexion axis. By studying 10 healthy subjects, they found that medial and lateral trochlear sizes could be the key parameters affecting the elbow joint flexion function, highlighting the potential for designing custom implants to improve joint function. Wen et al. explored the relationship between bearing extrusion and postoperative persistent pain in Oxford unicompartmental knee arthroplasty. The study evaluated the biomechanical factors that contribute to bearing extrusion and postoperative pain in patients with knee arthroplasty, highlighting the improvement in bearing movement trajectory in potential beneficial treatment of knee disorders. Wang et al. investigated the biomechanical relationship between posterosuperior rotator cuff tear (PSRCT) size and shoulder abduction function using cadaveric shoulders. These biomechanical testing suggested that the weight-bearing ability of the shoulder significantly decreased as PSRCT progressed. Chang et al. compared the muscle activation patterns and spinal kinematics between the hand-foot kneeling (HFK) position used in the traditional Chinese exercise Wuqinxi and the four-point hand-knee kneeling (HKK) position commonly used for core stabilization exercises. This study suggested that HFK was more effective for strengthening abdominal muscles, while HKK was more effective for strengthening lumbar muscles and increasing spine mobility, providing evidence for selecting specific exercises and developing individualized training programs.

Another exciting area of research that is highlighted in this Research Topic is the use of novel materials and technologies for the treatment of musculoskeletal disorders. For instance, Orth et al. evaluated the use of novel mineral-coated microparticles for spatiotemporal controlled delivery of VEGF and BMP-2, which showed great potential to improve bone healing in atrophic non-unions by promoting angiogenesis and osteogenesis as well as reducing early osteoclast activity. Ran et al. described a new method for bone preparation in total knee arthroplasty using an ultra-pulsed CO_2_ laser osteotomy system, which was shown to preserve natural bone structure and improve cell adhesion compared to traditional mechanical saws. This study suggested the promising application of ultra-pulsed CO_2_ laser in total knee arthroplasty (TKA) bone preparation, offering non-invasive bone cutting and long-term biological fixation. Besides these original articles, Wang et al. reviewed the application of iron oxide nanoparticles for bone regeneration in recent years, and outlined the mechanisms of iron oxide nanoparticles in bone tissue regeneration in detail based on the physicochemical properties, structural characteristics and safety of iron oxide nanoparticles. This review demonstrated the potential of these nanoparticles in enhancing bone growth and regeneration, highlighting the importance of biochemical regulation in the treatment of bone disorders.

Biochemical regulation, on the other hand, is controlled by various hormones, signaling pathways and growth factors, including VEGF, BMP-2, P1NP, and HbA1c. These substances play a crucial role in regulating the growth, development, and maintenance of the musculoskeletal system. For instance, Zheng et al. demonstrated that activating the Sirt1-autophagy signaling network through the pulsed electromagnetic field (PEMF) therapy can alleviate intervertebral disc degeneration, thereby protecting extracellular matrix (ECM) and reducing intervertebral disc (IVD) degeneration. This finding highlights the critical role of Sirt1-dependent autophagy signaling pathway in ECM protection, and the establishment of therapeutic effect of PEMF on IVD degeneration.

Recent research has focused on the interplay between biomechanical and biochemical regulation of the musculoskeletal system. It has been discovered that mechanical loading can influence the expression of certain genes that regulate muscle growth and repair. Furthermore, biochemical signals can also affect the biomechanical properties of the musculoskeletal system, including the stiffness and strength of bone and connective tissue. For example, Jia et al. focused on the prediction of femoral strength based on bone density and biochemical markers in elderly men with type 2 diabetes mellitus, which illustrated the interplay between biomechanical and biochemical regulation in the treatment of musculoskeletal disorders.

Another fascinating aspect of this Research Topic is the use of computational models to gain insight into the biomechanical and biochemical regulation of the musculoskeletal system. Jeong et al. evaluated the optimal surgical plan for the treatment of extraocular muscle damage in thyroid eye disease patients based on finite element analysis. This study highlighted the potential benefits of using finite element model in designing surgical interventions to reduce damage to the extraocular muscle during treatment strategies. Similarly, Jia et al. focused on the prediction of femoral strength based on bone density and biochemical markers in elderly men with type 2 diabetes mellitus. This study showed how computational models can be used to assess the risk of bone fractures and guide clinical decision-making. Wang et al. investigated the biomechanical effects of medial meniscus radial tears on the knee joint during gait using a finite element model. This study demonstrated that radial tears with larger widths could lead to high stress concentrations, contributing to a better understanding of meniscal tear-induced biomechanical changes during human activities and potential surgical guidance of meniscectomies and the prophylaxis and treatment of OA. Fang et al. used computational kinematic simulations to investigate the effect of different rotational alignments of the tibial component on knee kinematics after total knee arthroplasty. The results showed that moderate external rotation of the tibial component generated more natural knee kinematics than internal rotation or neutral position of the tibial component. Pei et al. investigated the kinematic and biomechanical effects of spinal distraction surgery in children with early onset scoliosis using 3D finite element analysis. This study revealed that traditional bilateral fixation can cause spinal re-imbalance and unexpected cervical lordosis and lateral displacement, and that more attention should be paid to spinal kinematic and biomechanical changes, providing a better understanding of spinal distraction surgery. Wu et al. proposed a principal-strain criterion to evaluate the safety of the fixation construct in cervical kyphosis correction and validates it against a retrospective case of anterior cervical discectomy fusion (ACDF), using finite element model. This study suggested that the ACDF restricted the ROM of cervical segments and lent stability to vertebra fusion, and the shape of the anterior cervical plate conforming to the curvature of the vertebra and screws fully inserted into vertebrae reduced the deformation concentration around the screw trajectories. Zeng et al. used finite element analysis to investigate the stability of internal fixation systems in different subtypes of Schatzker II fracture of the tibial plateau, and found that the use of fillers at the defect site can effectively reduce stress concentration of the implant and loss of the collapsed block, providing good stability for the fracture. This study highlighted finite element analysis as a powerful tool to help improve clinical outcomes.

In conclusion, the articles in this Frontiers Research Topic provide valuable insights into the biomechanical and biochemical regulation of the musculoskeletal system. The studies highlight the mechanical, biochemical factors, and the complex interplay between them in the maintenance of musculoskeletal health and the development of musculoskeletal disorders. The findings of these studies have important implications for the development of new treatments and therapies for musculoskeletal disorders, as well as for the design of prosthetics and orthotics. The use of novel materials, technologies, and computational models is also an exciting area of research that has the potential to revolutionize the field of musculoskeletal biomechanics. Overall, the articles in this Research Topic represent a significant contribution to the field of bioengineering and biotechnology and will be of great interest to researchers, clinicians, and engineers working in this area. The primary challenges within the field of Biomechanical and Biochemical Regulation of the Musculoskeletal System involve deciphering the mechanisms behind musculoskeletal injuries and diseases, enhancing prevention and treatment approaches, and investigating the interplay between mechanical forces and biochemical signaling pathways. In order to tackle these Research Topic, future studies will employ cutting-edge imaging, computational modeling, molecular biology methods, clinical trials, and interdisciplinary partnerships.
